# Supplementary material for: Learning curve and surgical outcome of robotic assisted colorectal surgery with ERAS program
Source: Sci Rep. 2022 Nov 29;12:20566. doi: 10.1038/s41598-022-24665-w (PMC9709162; doi:10.1038/s41598-022-24665-w)
Supplement: Supplementary file 1 — Supplementary Information 1. [file 41598_2022_24665_MOESM1_ESM.zip › LSC Raw data-Ñ[▒K/103/H_ñw╝╨░O▒Kñσ.pdf]

正式摘要(電子簽章：000000203249093)

W55 / W55 ☐英文標題(注意:無英文姓名-請補病人基本檔!) ☐【讀取】憑證

臺中榮民總醫院
出院病歷摘要

轉入號： 轉出號：

[redacted]

(8)流水編號	
(9)入院日期	109/11/11 (2020) 直腸外科 W55 - 263
(10)轉科日期	
(11)出院日期	109/11/16 (2020) 住院天數計 5 日

診斷

- 入院：R-S colon cancer
  DM
  HTN
  CVA twice under plavix with Rt hemiparesis and dysphasia
- 出院：rectal cancer, s/p laparoscopic LAR on 20201112
  DM
  HTN
  CVA twice under plavix with Rt hemiparesis and dysphasia

主訴

- Blood in stool and tenesmus for half year.

病史

- This is a 77 y/o man with history of HTN,DM, CVA twice under plavix with Rt hemiparesis and dysphasia, who presented with blood in stool and tenesmus for half year. He first went to 沙鹿光田醫院 where colonoscopy on 2020/11/05 revealed suspected R-S colon cancer at 15cm from FAV with biopsy. He then went to our SER and then OPD. This time, he is admitted for operation.

體檢發現

-
  - General appearance :
    - easy looking , conscious clear , without cardiopulmonary distress
    - Vital signs自動帶入date 體溫(BT) 脈搏(P) 呼吸(R) 血壓(BP) 身高(BH) 體重(BW) BMI
      20201111 36℃ 82次/分 18次/分 173/71mmHg 164.7cm 72kg 26.5
  - HEENT :
    - Eyes: no anemic conjunctiva, no icteric sclera, normal EOM
    - ENT: no otorrhea, no rhinorrhea, no erythematous mucosa, no injected tonsil, no deformity
  - Neck: supple, freely movable, trachea midline,no deformity, no engorged jugular vein , no palpable thyroid or lymph node enlargement
  - Chest:
    - Inspection: Grossly normal, symmetric and fully expansion, no accessory muscle used, no operation scar, no spider angiomata, no gynecomastia
    - Palpation: no palpable mass or nodule, no subcutaneous emphysema, no tenderness
    - Ausculation: clear breathing sound
  - Heart:
    - Percussion: normal heart size
    - Ausculation: regular heart beat, normal S1, S2, no gallop, no murmur
  - Abdomen:
    - Inspection: in
      - flat contour, symetrically, no scar, no stria, no dilated veins, no rashes

-
  - Ausculation: normoactive bowel sound, no abdominal bruits
  - palpation: soft, no muscle guarding or rigidity, no local tenderness, no rebounding pain, no palpable mass, no palpable pulsation
  - Percussion: no shifting dulllness, no hepatomegaly, no splenomagaly
-

Peripheral pulsation :	CA	BA	RA	FA	PA	PTA	DPA
Right side :	++	++	++	++	++	++	++/+++
Left side :	++	++	++	++	++	++	++/+++

- Genito-anus: no tenderness or palpable mass lesion or rectal shelf, normal external genitalia, normal anal tone, brown stool

CRS CONDITION

-
  - Lung

手術日期及方法

- 手術日期及方法20201112 1. Laparoscopic low anterior resection, end-to-end anastomosis, EndoGIA 60/.38mm, CEEA- 29mm. Bilateral doughnut: intact, air tight test: done, no air leak.
  2. High ligation (+) above the left colic artery.
  3. Cut margin: proximal 15 cm, distal 3 cm.
  5. Take down of splenic flexure: (-).
  6. No drain placed.

住院治療經過

- After admission, operation was done on 11/12. Try clear liquid diet on 11/12. Try semiliquid diet on 11/14. Due to relative stable condition, MBD was permitted and OPD f/u is scheudled for further treatment.

合併症

一般檢查

特殊檢查

病理報告

其它

出院時情況

- 15.計劃性出院

出院指示

藥名	劑量	劑量單位	用法	頻次	數量	數量單位	現況	天數	單位
Ultracet tab	1	TAB	PO	Q6H	32	TAB	TH	8	天
4mg Doxaben XL tab	1	TAB	PO	QD	8	TAB	TH	8	天
BethanechoL tab 25mg-JS	1	TAB	PO	TIDAC	24	TAB	TH	8	天

出院計劃

主治醫師 2062F 蔣鋒帆 / 住院醫師 2310K 陳瑋夫

正式摘要(電子簽章：000000203249093)

W55 / W55

doctorRank:B
